# High Diversity of *Cryptosporidium* Subgenotypes Identified in Malaysian HIV/AIDS Individuals Targeting gp60 Gene

**DOI:** 10.1371/journal.pone.0031139

**Published:** 2012-02-08

**Authors:** Asma Iqbal, Yvonne A. L. Lim, Johari Surin, Benedict L. H. Sim

**Affiliations:** 1 Department of Parasitology, Faculty of Medicine, University of Malaya, Kuala Lumpur, Malaysia; 2 Bureau of Microbial Hazards, Health Canada, Banting Research Centre, Ottawa, Ontario, Canada; 3 Infectious Disease Unit, Department of Medicine, Hospital Sungai Buloh, Sungai Buloh, Selangor Darul Ehsan, Malaysia; Instituto de Higiene e Medicina Tropical, Portugal

## Abstract

**Background:**

Currently, there is a lack of vital information in the genetic makeup of *Cryptosporidium* especially in developing countries. The present study aimed at determining the genotypes and subgenotypes of *Cryptosporidium* in hospitalized Malaysian human immunodeficiency virus (HIV) positive patients.

**Methodology/Principal Findings:**

In this study, 346 faecal samples collected from Malaysian HIV positive patients were genetically analysed via PCR targeting the 60 kDa glycoprotein (gp60) gene. Eighteen (5.2% of 346) isolates were determined as *Cryptosporidium* positive with 72.2% (of 18) identified as *Cryptosporidium parvum* whilst 27.7% as *Cryptosporidium hominis*. Further gp60 analysis revealed *C. parvum* belonging to subgenotypes IIaA13G1R1 (2 isolates), IIaA13G2R1 (2 isolates), IIaA14G2R1 (3 isolates), IIaA15G2R1 (5 isolates) and IIdA15G1R1 (1 isolate). *C. hominis* was represented by subgenotypes IaA14R1 (2 isolates), IaA18R1 (1 isolate) and IbA10G2R2 (2 isolates).

**Conclusions/Significance:**

These findings highlighted the presence of high diversity of *Cryptosporidium* subgenotypes among Malaysian HIV infected individuals. The predominance of the *C. parvum* subgenotypes signified the possibility of zoonotic as well as anthroponotic transmissions of cryptosporidiosis in HIV infected individuals.

## Introduction

The world is currently plagued with a global pandemic of human immunodeficiency virus (HIV) infection. From its discovery in 1981 to 2010, acquired immunodeficiency syndrome (AIDS) has killed more than 34 million people worldwide. At present, HIV infects about 0.5% of the world's population [1]. HIV epidemic has expanded rapidly in Malaysia recording a cumulative 105,471 HIV/AIDS cases in 2010 [2] since its first case in 1987 [3]. HIV-infected individuals in developing countries such as Malaysia are susceptible not only to opportunistic infections but also predisposed to a myriad of enteric pathogens which are endemic in the tropics [4]. Reports from many regions of the world where HIV/AIDS is endemic have also acknowledged that intestinal parasitism is widespread among these populations [5].

One of the most common opportunistic intestinal parasite is *Cryptosporidium*, a causative agent of cryptosporidiosis, a disease which is commonly found in HIV-infected individual and is currently listed as an AIDS defining illness by US Centers for Disease Control and Prevention [6]. Globally the prevalence rate of *Cryptosporidium* infection may account for 10 to 20% of the cases of diarrhea in HIV-infected patients living in developed countries and as much as 50% in under privileged countries [7], [8].

In Malaysia, the first cryptosporidiosis case was reported in 1984 [9]. Subsequently, there were five reports with prevalence ranging from 3% to 23% among HIV-infected individuals [4], [10], [11], [12], [13]. Two studies have noted the preponderance of cryptosporidiosis among HIV patients with CD4 count less than 200 cell/mm^3^ [4], [12]. With impaired immunity especially in patients with low immune level (CD4 counts <200 cells/mm^3^), infections with intestinal parasites may result in diarrheal symptoms [14]. Patients with CD4 count of >180 cells/mm^3^ usually have self-limiting infections, whereas most patients with counts <140 cells/mm^3^ develop severe and persistent infections [15]. With the introduction of HAART which partially restores the immune function, the incidence of opportunistic parasite infection such as cryptosporidiosis has declined [16]. In Malaysia, where infections with *Cryptosporidium* is widespread [17], [18] and a large proportion of HIV/AIDS patients are still not receiving HAART, it is crucial that efforts are made to improve the understanding of this opportunistic parasite in such patients [12], in particular the genetic makeup of the parasite.

There is extensive genetic variation within the genus *Cryptosporidium*. With at least 23 species of *Cryptosporidium* being considered valid by most investigators, these species are collectively found in human, mouse, cattle, pig, sheep, horse, goat, cat, dog, kangaroo, chicken, turkey, fish, ferrets, lizard, monkey and deer [19], [20]. With a few exceptions, most species and genotypes are host-adapted in nature, having a narrow spectrum of natural host. Of these 23 species, at least 7 have been found to infect HIV-infected individuals (i.e. *C. parvum*, *C. hominis*, *C. meleagridis*, *C. felis*, *C. muris*, *C. canis* and *C. suis*). The two species of greater significance in terms of public health, however, are *C. parvum* and *C. hominis*. Given the wide range of *Cryptosporidium* species, genotypes, and subgenotypes infecting humans, and each may have different sources of infection, transmission routes, and pathogenicity [21], [22], identifying the species and genotypes present in a population is crucial for the identification of associated factors for transmission and implementing control programs to limit exposure to infectious oocysts.

Recent advances in the molecular characterization of *Cryptosporidium* parasites have made it possible to differentiate *Cryptosporidium* oocysts at species, genotypes and subgenotypes levels [23]. One such tool is based on sequence analysis of the 60-kDa glycoprotein gene (gp60), which allows the identification of many genotype families and subgenotypes within each one [24]. Currently, the gp60 gene is the most suitable and widely used genetic marker for *Cryptosporidium* species infecting humans [25]. This locus is useful for such studies because it contains multiple regions displaying high mutation rates, including in particular, a “hyper-variable” microsatellite region [26]. Understanding the subgenotypes of *C. hominis* and *C. parvum* may provide clues into the mechanisms of infection of these organisms and lay scientific foundations for effective therapeutic modalities [27], [28].

Generally, there are eleven gp60 genotype families found in *C. parvum* [25]. Genotype families IIa and IId are zoonotic as these two have been identified infecting humans and animals. Genotype families IIc and IId are also common and broadly distributed [25]. Presently, *C. hominis* comprises six defined gp60 genotype families (Ia–Ig; excluding Ic, which is yet undefined) [25].

Studies of the gp60 gene have shown that certain genotype families are geographically related, commonly found genotype families Ia, Ib, Id and Ie which are commonly seen in Kenya, Malawi, India, Peru and USA [24], [26], [29], [30], [31], [32]. Although cryptosporidiosis is prevalent in developing countries, genetic characterization is lacking in Asia, especially in Malaysia. Subgenotyping studies have only been conducted in India [33], [34], [35] and very recently in Malaysia [12]. Therefore, the aim of the present study was to further expand the previous study by our group [12] in order to determine the diversity of subgenotypes of *Cryptosporidium* isolated from hospitalized HIV infected individuals from Malaysia in analyzing the polymorphisms at the gp60 locus.

## Material and Methods

### Sample collection

Fecal samples were collected from 346 HIV-infected individuals from three different hospitals in Malaysia, namely: Hospital Sungai Buloh, Selangor; University of Malaya Medical Centre, Kuala Lumpur and Hospital Raja Zainab Perempuan II, Kelantan. Ethical clearance from the Medical Ethics Committee of University Malaya Medical Centre (IRB Ref. No. 655.17) and Ministry of Health National Medical Research Register (MOH-NMRR) (MOH-NMRR ID: # 09-286-3930) were obtained prior to the commencement of the study.

Informed consent was obtained from each HIV-infected patient who voluntarily participated after a clear explanation of the research objectives. The inclusion criteria for participation were HIV infected patients regardless of age, race, with or without symptoms and patients who consented to the study, whereas the exclusion criteria were those who were not HIV infected and who did not give their consent to participate in the study. Clinical data were obtained from patient's medical record with patient's consent and permission from health authorities. Fecal samples were preserved in 2.5% potassium dichromate solution and stored at 4°C for further analysis [36].

Clinical data were available only for 253 patients out of 346 (93 patients did not provide consent for their clinical records to be accessed). In addition, of the 253 HIV patients whose clinical data were available, there were 130 (51.4% of 253) patients who also had opportunistic infections (OIs), whereas 123 (48.6%) did not have OIs.

### Extraction of *Cryptosporidium* genomic DNA

#### Purifying stool samples with immunomagnetic separation method

A total of 346 stool samples were used for molecular characterization. The oocyst samples were purified by IMS according to the manufacturer's instructions in the Dynal IMS Kit from Dynal GC-Combo kit (Dynal, cat. No. 730.02, Oslo, Norway). Briefly, 10 ml of sample suspension was transferred into tube which has been added with 1 ml of 10× SL buffer A and buffer B, then 100 µl of Dynabeads anti-*Cryptosporidium* was added into the tube. The tube was affix to a rotating mixer and was rotated at approximately 18 rpm for 1 hour at room temperature. The sample tube was removed from the mixer and placed in the magnetic particle concentrator (MPC-1). The sample was then rocked gently for 2 minutes, all supernatant decanted from the tube. After that, the capture tube was removed from MPC-1 and the sample was resuspended in 1 ml of 1× SL Buffer A. The mixture was then transferred into a 1.5 ml microcentrifuge tube. The microcentrifuge tube was placed into the second magnetic particle concentrator (MPC-S) with magnetic strip in place. The tube was gently rocked / rolled for 1 minute. Without removing the tube from MPC-S, the supernatant was gently aspirated from the tube. The oocysts-beads complex was then stored in −20°C.

### DNA extraction

Genomic DNA was extracted from the IMS-isolated oocysts using QIAgen DNA Mini kit (QIAGEN, cat. No. 51306, Germany) according to manufacturer's description. Briefly, IMS-isolated oocysts were resuspended in lysis buffer and underwent 5 consecutive cycles of freezing in liquid nitrogen for 1 min and thawing at 56°C for 2 min, with vortexing for 30 sec for every cycle in order to rupture the *Cryptosporidium* oocysts. DNA was eluted in 50 µ l buffer (Qiagen) and stored at −20°C until further use.

### Amplification of DNA by nested-PCR targeting gp60 gene

Two step nested-PCR assays [26], [37] were used to generate amplicons for subgenotyping by sequencing a fragment of the gp60 gene. In the first round of PCR, the gp60 gene (∼980–1000 bp) was amplified from DNA using a 21-mer forward primer (gp15-ATG; 5′-ATG AGA TTG TCG CTC ATT ATC-3′) and a 21-mer reverse primer (gp15-STOP; 5′-TTA CAA CAC GAA TAA GGC TGC-3′). Subsequently, a 450-bp fragment was amplified in the secondary reaction using primers gp15-15A (5′-GCC GTT CCA CTC AGA GGA AC -3′) and gp15-15E (5′-CCA CAT TAC AAA TGA AGT GCC GC -3′). The primary reaction was prepared in a total volume of 50 µl in 0.2 ml tubes consisted of premixed reagents containing 200 µM of each of the four deoxynucleoside triphosphates (dNTP) (Fermantas, cat. No. #R0192, Ontario, Canada), 4 µM of each of primers gp15-ATG/ gp15-STOP and gp15-15A/ gp15-15E (Bio-Basic, Canada), 3 mM MgCl_2_ (Fermentas, cat.no. # R0971, Ontario, Canada), 2.5 U *Taq* polymerase (New England Biolabs, cat. No. M0267L, Ipswich, USA), and 1× ThermoPol PCR buffer (New England Biolabs, cat. No. M0267L, Ipswich, USA). Two µl of DNA template was used in the primary PCR whereas 5 µl of the first PCR product was used as template in the secondary PCR. The secondary PCR reagent concentrations were similar to the primary as mentioned above. The cycling condition was as follows; hot start at 94°C for 5 min, followed by 40 cycles of denaturing for 30 sec at 94°C, annealing for 45 sec at 55°C and extension for 1 min at 72°C, followed by a final extension at 72°C for 10 min. The secondary PCR had a 35 cycle conditions and the annealing time and extension time in the secondary PCR was 30 sec instead of 45 sec and 1 min, respectively. The PCR products were resolved on 2% agarose gel and visualized with Sybr safe stain.

### Purification of PCR product

The PCR product was purified using QIAquick PCR purification kit (QIAgen, cat. No. 28104, Germany) according to the manufacturer's protocol. The volume of elution buffer added to the column was 30 µl to increase the concentration of the eluted DNA.

### DNA sequencing

For the *Cryptosporidium* isolates of gp60 gene, sequencing was done in both directions using forward primer (gp15-15A; 5′-GCC GTT CCA CTC AGA GGA AC -3′) and reverse primer (gp15-15E; 5′-CCA CAT TAC AAA TGA AGT GCC GC -3′) [28]. DNA sequencing was carried out by Medigene (SOLGENT CO. LTD, Korea).

### Sequence analysis and phylogenetic analysis

Nucleotide sequences for all 18 gp60 subgenotypes were obtained directly from the nested PCR amplicons and sequenced in forward and reverse directions. All 18 pairs of sequences were edited via Applied Biosystems Sequence Scanner software v1.0 Sequence Trace Viewer and Editor (www.en.bio-soft.net/dna/ss). Edited sequences were aligned and consensus sequences were created for each isolates using the BioEdit (www.mbio.ncsu.edu) programme. Each consensus sequence was used for the identification of *Cryptosporidium* gp60 genotypes and sequences were searched using basic local alignment search tool (BLAST) (www.ncbi.nlm.nih.gov/blast) in order to get the 100% similarity with the gp60 genotypes sequences deposited in the GenBank and published in peer-reviewed international scientific journals. Gp60 sequences were compared with reference sequences of *C. parvum* and *C. hominis* families. Isolates of *C. parvum* genotypes IIa (HQ631411, HQ631412, HQ631413, HQ631414, HQ631415, HQ631416, HQ631417, HQ631418, HQ631419, HQ631420, HQ631421 and HQ631422) and *C. parvum* genotypes IId (HQ631423) sequences together with other *C. parvum* genotypes families reference sequences (i.e., IIa, IIb, IIc, IId, IIe, IIf, IIg, IIh, IIi, IIj and IIk) together with *C. hominis* (AY738187) [24] as outgroup were aligned via the program Clustal W [38]. Multiple sequence alignment of the representative *Cryptosporidium* isolates and reference sequences of *C. parvum* were conducted using the program BioEdit [39]. Whereas, isolates of *C. hominis* genotypes Ia (HQ631406, HQ631407 and HQ631408), and *C. hominis* genotypes Ib (HQ63109 and HQ631410) sequences collectively with similar *C. hominis* genotypes families reference sequences (i.e., Ia, Ib, Id, Ie and If) together with *C. parvum* (EU164809) [40] as outgroup were aligned via the program Clustal W [38] and multiple sequence alignment were generated by using the BioEdit programme [39].

The phylogenetic analysis of the gp60 gene nucleotide sequence data were conducted via Bayesian Inference using the software package MrBayes v3.1.2 (http://mrbayes.csit.fsu.edu/index.php). Upon the completion of the Bayesian analysis, a 50% majority-rule consensus tree for each species and genotypes were constructed in Treeview X v.0.5. (http://darwin.zoology.gla.ac.uk/_rpage/treeviewx/). The percentage of replicate trees in which the associated taxa clustered together in the bootstrap test values (1.00) is shown next to the branches.

### Microsatellite analysis of *Cryptosporidium* gp60 subgenotypes isolated from HIV patient

All the 18 gp60 *Cryptosporidium* sequences were analyzed for “TCA” microsatellite region. Gp60 subgenotype results analysis display high mutation rates, in particular, a “hyper-variable” microsatellite region. The gp60 subgenotype “TCA” microsatellite region, showed triplet codons were categorized according to the number of trinucleotide repeats coding for the amino acid serine. *Cryptosporidium* gp60 subgenotypes consist of a variable number of “A” (TCA), “G” (TCG), “T” (TCT) and “R” (ACATCA) [40], [41], [42].

### Nucleotide sequence accession number

Data on the nucleotide sequences of the gp60 gene of *C. parvum* genotypes have been deposited in GenBank under the accession numbers HQ631411 to HQ631423 representing isolates of *C. parvum* genotypes IIa and IId respectively. Whereas, *C. hominis* have been deposited in GenBank under the accession numbers HQ631406 to HQ631410 representing isolates of *C. hominis* genotypes Ia and Ib. *C. parvum* genotype IIa accession number HQ631423 and *C. hominis* genotype Ia and Ib with accession numbers HQ631406, HQ631408, HQ631409 and HQ631410 have also been reported in another publication by our group [12].

## Results

**Figure 1:**
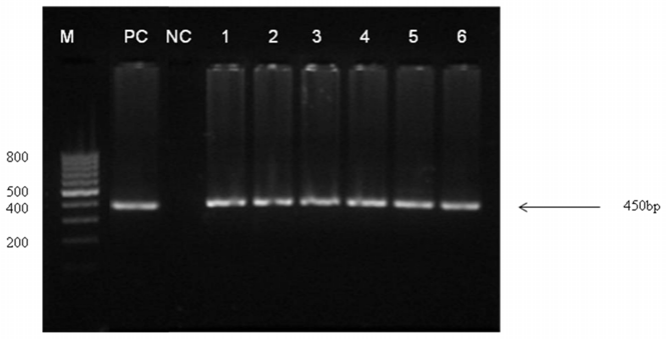
Representative presentation of PCR products generated from *Cryptosporidium* gp60 gene from fecal specimens on 2% agarose electrophoretic gel. M: 100-bp DNA marker, with molecular size indicated in base-pair unit for prominent band. NC: Negative control/DNA blank (PCR mixture plus the product of the first negative control which was used in the primary PCR as template). PC: Positive control (*Cryptosporidium parvum* H3 isolate). Lanes 1–6: Secondary PCR product from *Cryptosporidium* positive specimens.

Analysis of *Cryptosporidium* via nested-PCR targeting 60 kDa glycoprotein (gp60) gene resulted in a distinct band at 450 bp (Figure 1). Eighteen (5.2% of 346) isolates were determined as *Cryptosporidium* positive with 72.2% (of 18) identified as *C. parvum* whilst 27.7% as *C. hominis*. Of these 18 *Cryptosporidium* infected HIV patients, personal and clinical information were also obtained. Most patients infected with *Cryptosporidium* were male (88.8%, 16 of 18), >35 years of age (mean age 38.6 yrs, 55.5%), asymptomatic (94.4%) and had a CD4 count of <200/mm^3^ (55.5%). There were two patients (11.1%) who had CD4 counts >200 cells/mm^3^ while six (33.3%) patient's CD4 counts were not available from their clinical records.

BLAST results of the gp60 positive samples showed that HIV patients harboured four *Cryptosporidium* genotypes, 13 (72.2%) were identified as *C. parvum* genotypes IIa and IId and 5 (27.7%) were *C. hominis* genotypes Ia and Ib (Table 1).

**Table 1:** *Cryptosporidium* subgenotypes and microsatellite analysis of gp60 gene in HIV patients (n = 18).

Species	No. of Patients	Genotypes	Subgenotypes	No. of Isolates
*Cryptosporidium parvum*	13 (72.2%)	IIa	A13G1R1	2
			A13G2R1	2
			A14G2R1	3
			A15G2R1	5
		IId	A15G1R1	1
*Cryptosporidium hominis*	5 (27.7%)	Ia	A14R1	2
			A18R1	1
		Ib	A10G2R2	2

**Figure 2:**
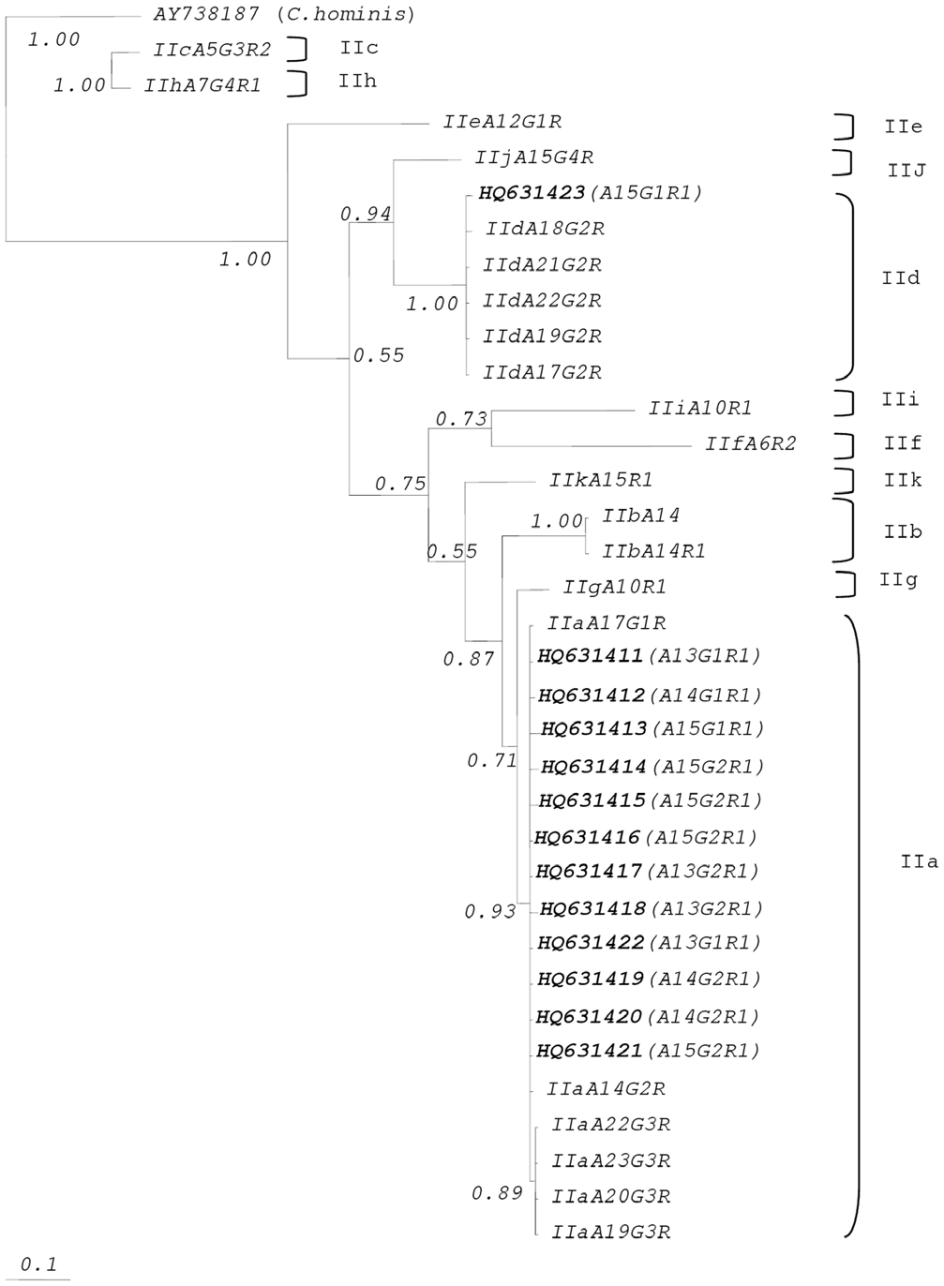
Phylogenetic analysis of gp60 sequence data representing *Cryptosporidium parvum* from HIV patients using Bayesian inference (BI). Sequences from the present study as well as reference sequences representing *C. parvum* subtypes (acquired from GenBank) are indicated. Posterior probabilities are indicated at all major nodes. *C. parvum* genotype IIa accession number HQ631423 have been reported in another publication by our group [12].

**Figure 3:**
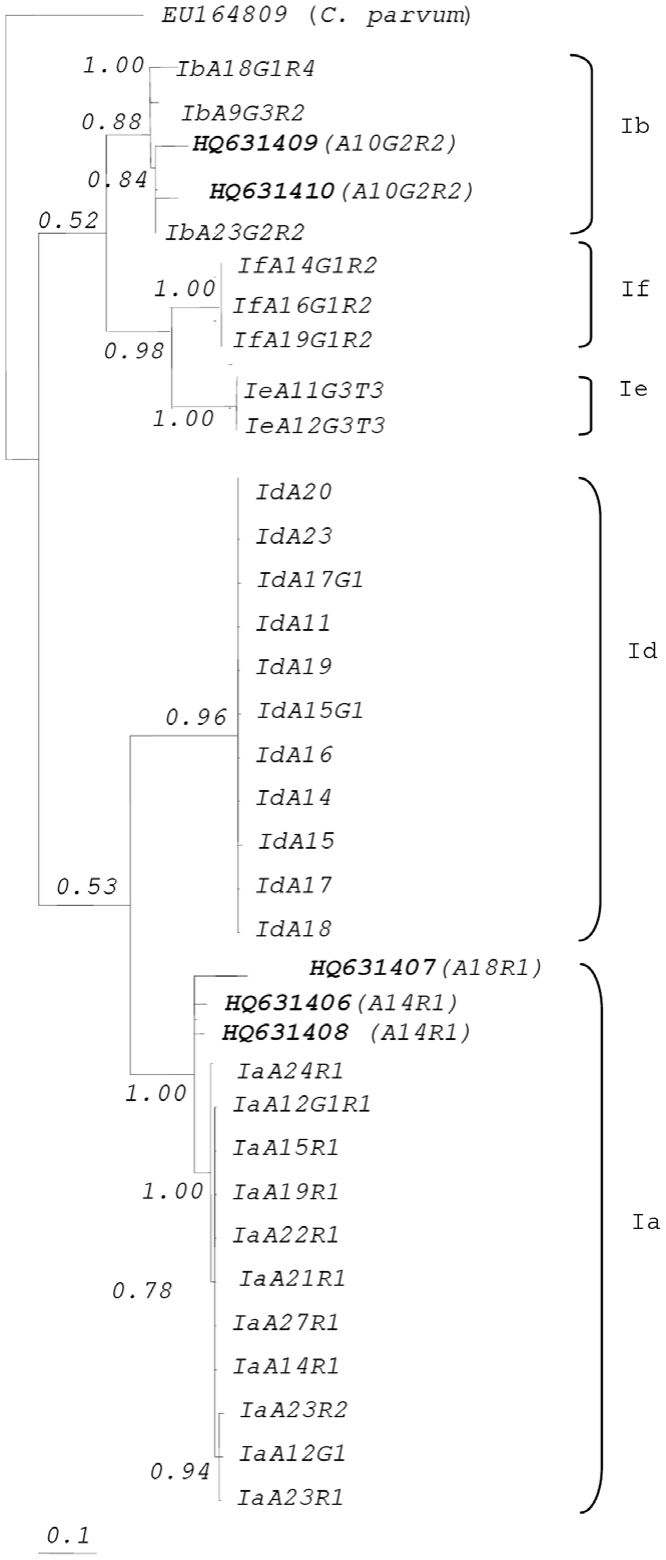
Phylogenetic analysis of gp60 sequence data representing *Cryptosporidium hominis* from HIV patients using Bayesian inference (BI). Sequence from the present study as well as reference sequences representing *C. hominis* subtypes (acquired from GenBank) are indicated. Posterior probabilities are indicated at all major nodes. *C. hominis* genotype Ia accession numbers HQ631406 and HQ631408 and genotype Ib accession numbers HQ631409 and HQ631410 have been reported in another publication by our group [12].

Phylogenetic analysis of sequence data of gp60 *C. parvum* genotypes IIa and IId isolates of HIV patients were conducted by Bayesian Interferance (BI) (Figure 2). The relationship of gp60 genotypes of *Cryptosporidium* isolates and reference sequences of *C. parvum* genotypes recognized the presence of genotypes IIa and IId in HIV patients. *Cryptosporidium* isolates of *C. parvum* genotypes IIa (HQ631411, HQ631412, HQ631413, HQ631414, HQ631415, HQ631416, HQ631417, HQ631418, HQ631419, HQ631420, HQ631421 and HQ631422) were clustered with reference sequence of *C. parvum* genotypes IIa (A14G2R1, A17G1R1, A19G3R1, A20G3R1, A22G3R1 and A23G3R1) with high internode value (i.e., 0.93). Whilst isolate of *C. parvum* genotypes IId (HQ631423) was grouped with reference sequences of *C. parvum* genotypes IId (A17G2R1, A18G2R1, A19G2R1, A21G2R1, A22G2R1) with highest internode value (i.e., 1.00). Analysis of the gp60 *C. hominis* genotypes Ia and Ib isolates of HIV patients were built by means of Bayesian Interferance (BI) (Figure 3). The association of gp60 genotypes of *Cryptosporidium* isolates and reference sequences of gp60 *C. hominis* genotypes predicted the presence of genotypes Ia and Ib in this study. *Cryptosporidium* isolates of *C. hominis* genotypes Ia (HQ631406, HQ631407 and HQ631408) were grouped with reference sequences of *C. hominis* genotype Ia (A12G1, A12G1R1, A14R1, A15R1, A19R1, A21R1, A22R1, A23R1, A23R2, A24R1 and A27R1) with high internode value (i.e., 1.00). Furthermore, isolates of *C. hominis* genotype Ib (HQ631409 and HQ631410) were clustered with reference sequences of *C. hominis* genotype Ib (A9G3R2, A18G1R4, A23G2R2) with 0.88 internode value.

Microsatellite analysis revealed that in the present study, *C. parvum* gp60 genotype found in HIV patients comprised of genotypes IIa and IId. *C. parvum* genotype IIa included two isolates of subgenotypes IIaA13G1R1 and IIaA13G2R1 each, three isolates of IIaA14G2R1 and five isolates of IIaA15G2R1. Whilst *C. parvum* IId consisted of a single isolate of subgenotype IIdA15G1R1. The present results disclosed that the majority of *C. parvum* IIaA15G2R1were male, Malay, IVDU having CD4 cells count <200 cells/mm^3^ (Table 2). These patients had co-infection with OIs such as *Mycobacterium tuberculosis* infections, toxoplasmosis, candidiasis, salmonellosis, cryptococcosis and histoplasmosis. Interestingly, a Chinese male, IVDU patient with subgenotype IIaA15G1R1 had CD4 cells count >200 cells/mm^3^ (i.e., 320 cells/mm^3^).

**Table 2:** Clinical data on *Cryptosporidium* infected HIV patients and the genetic analysis of gp60 gene.

Sample no.	Age (yrs.)	Sex^a^	Ethinc race^b^	CD4 count (10^3^/ml)	Mode of transmission^c^	Species	Genotype p*gp60*	Subgenotype	Opportunistic infections ( OIs)
1	31	M	M	6	IVDU	*C. parvum*	IIa	A13G1R1	Histoplasmosis, Salmonella septicemia
2	46	M	M	3	IVDU	*C. parvum*	IIa	A14G2R1	Cerebral Toxoplasmosis
3	31	M	M	34	Unknown	*C. parvum*	IIa	A15G2R1	Cerebral Toxoplasmosis
4	51	M	C	320	IVDU	*C. parvum*	IIa	A15G2R1	No OIs
5	36	M	C	2	IVDU	*C. parvum*	IIa	A15G2R1	Candidiasis
6	38	M	M	27	IVDU	*C. parvum*	IIa	A15G2R1	Mycobacterium tuberculosis
7	41	M	M	75	Heterosexual	*C. parvum*	IIa	A13G2R1	Cryptococcosis
8	42	M	M	59	IVDU	*C. parvum*	IIa	A13G2R1	No OIs
9	13	M	M	NA	NA	*C. parvum*	IIa	A14G2R1	NA
10	1	F	M	NA	NA	*C. parvum*	IIa	A14G2R1	NA
11	47	M	I	NA	IVDU	*C. parvum*	IIa	A15G2R1	NA
12	32	M	M	NA	NA	*C. parvum*	IIa	A13G1R1	NA
13	39	F	I	NA	NA	*C. parvum*	IId	A15G1R1	NA
14	46	M	C	10	Heterosexual	*C. hominis*	Ia	A14R1	*Herpes simplex*, Kaposi's sarcoma
15	22	M	M	38	IVDU	*C. hominis*	Ia	A18R1	No OIs
16	35	M	C	NA	NA	*C. hominis*	Ia	A14R1	NA
17	24	M	F	256	Heterosexual	*C. hominis*	Ib	A10G2R2	Cryptococcosis
18	42	M	C	2	IVDU	*C. hominis*	Ib	A10G2R2	Mycobacterium tuberculosis, Salmonella septicemia

a : M = male, F = female.

b : M = Malay, C = Chinese, I = Indian, F = Foreigner (Burmese),

c : IVDU = Interavenous drug users,

NA: Data not available.

Subsequently, *C. hominis* genotypes Ia and Ib encode the microsatellite triplet codons based on the integer of trinucleotide repeats. Subgenotype exploration showed that isolates of gp60 *C. hominis* genotype Ia were determined as two isolates of subgenotypes IaA14R1 and single isolate of subgenotypes IaA18R1 whereas, further analysis of *C. hominis* Ib indicated two isolates of subgenotype IbA10G2R2 (Table 1). Subgenotype IaA14R1 was identified in a heterosexual, Chinese male having CD4 counts <50 cells/ mm^3^ (Table 2). Other co-infections included *Herpes simples* and Kaposi's sarcoma. Whilst subgenotype IaA18R1 was found in a Malay IVDU who did not have any other opportunistic infection. However, *C. hominis* subgenotype IbA10G2R2 was discovered in two patients. One was an Indian IVDU with CD4 counts <50 cells/ mm^3^ and having OIs such as *Mycobacterium tuberculosis* infection and salmonellosis. Whilst the other was a female Burmese heterosexual with CD4 counts >200 cells/ mm^3^ and experiencing co-infection with cryptococcosis (Table 2).

## Discussion

In this study, using nested-PCR technique targeting the gp60 gene, it was discovered that 5.2% (18 of 346) of sampled Malaysian HIV individuals were infected with *Cryptosporidium*. BLAST analysis of these 18 sequences which was confirmed by phylogenetic analysis showed that 13 (72.2%) were of *C. parvum* genotype and 5 (27.7%) of *C. hominis* genotype. The *C. parvum* gp60 genotype found in these HIV patients comprised of genotypes IIa and IId. Within the *C. parvum* genotype IIa family, all sequences were identical in the non-repeat region (i.e. had one copy of sequence ACATCA immediately after the trinucleotide repeats), while the trinucleotide repeat region contained one and two copies of the TCG repeat and 13, 14 and 15 copies of TCA. *C. parvum* genotype IIa included 2 isolates of subgenotypes IIaA13G1R1 and IIaA13G2R1 respectively, 3 isolates of subgenotype IIaA14G2R1 and 5 isolates of subgenotype IIaA15G2R1. *C. parvum* gp60 subgenotype IIaA15G2R1 is the most common subgenotype detected in this study. *C. parvum* IId subgenotype consisted of a single isolate of IIdA15G1R. The present results indicated more diversity compared to previous results by our group, whereby in that study only subgenotype *C. parvum* IIdA15G2R1 was found [12].

Human infections with *C. parvum* genotype family IIa are commonly seen in areas with intensive animal production, such as the UK, Portugal, Slovenia, southeastern Australia and rural areas of the North America [28], [43]–[47]. This is partially due to relative high occurrence of zoonotic infections in these areas because cattle and sheep are commonly infected with *C. parvum* of the genotype family IIa, and some of the genotypes within the family have been found in both humans and animals in the same area [44], [45].

In contrast, *C. parvum* genotype IIa has been rarely found in humans in developing countries or cities in North America [32], [44], [48]. In the present study, *C. parvum* IIaA15G2R1 was found to be the most common subgenotype in the Malaysian HIV patients and this subgenotype has previously been widely reported from humans, calves and outbreaks linked to farms from various regions [28], [38], [46], [49], [50]–[53]. On the basis of these reports, we are suggesting that the transmission of *C. parvum* IIa among Malaysian HIV patients may be potentially zoonotic in nature.

Besides genotype IIa, *C. parvum* subgenotype IId (IIdA15G1R1) was also identified in the Malaysian HIV individuals. Genotype IId has been found in humans, AIDS patients and in cattle [25], [28]. Similarly, *C. parvum* genotype IId was commonly seen in Kuwaiti children [24]. Despite the high occurrence of IId in AIDS patients in Lisbon, IId infections in animals were only seen in the municipality of Odemira in southern Portugal [49]. As far as we know, four IId subgenotype have been recorded in both humans and non-human hosts; these are *C. parvum* IIdA17G2R1 [49], [54], IIdA19G2R1 [49], [55], IIdA22G2R1 [28], [55] (from humans and cattle) and IIdA18G2R1 [50], [56] (humans and goats), thus indicating that subgenotype IIdA15G1R1 found in the present study as the first subgenotype ever reported.

Presently, *C. hominis* comprises six defined gp60 subgenotype families (Ia–Ig; excluding Ic, which is yet undefined). The multiplicity of *C. hominis* subgenotype of the present study showed 5 (14%) of the 18 HIV-infected isolates being designated into two different genotypes (i.e., Ia and Ib). Microsatellite analysis indicated that isolates of gp60 *C. hominis* genotype Ia were determined as subgenotypes IaA14R1 in 2 isolates and IaA18R1 in 1 isolate whereas, further analysis of *C. hominis* genotype Ib indicated IbA10G2R2 subgenotype in two Malaysian HIV patients.

*C. hominis* tends to be responsible for most of human cryptosporidiosis in many regions of the world [57]. The distribution of gp60 subgenotype families in the world indicates the existence of population substructure in *C. hominis* [49]. *C. hominis* genotype Ib is a dominant and broadly distributed genotype whereas genotype Ia, Id and Ie are found less frequently as compared to genotype Ib [25]. *C. hominis* infections in AIDS patients from Portuguese indicated that two thirds of *C. hominis* parasites found in HIV-seropositive patients were of subgenotype IbA10G2 [49]. This strongly supported the results of the current study in which *C. hominis* IbA10G2R2 was found in the present studied HIV patients. *Cryptosporidium hominis* IbA10G2R2 has been identified also as the dominant subtype among isolates from humans infected during two waterborne outbreaks in Northern Ireland [43] and, more recently, a waterborne outbreak in France [58]. On the other hand, IbA10G2R2 is the only *C. hominis* gp60 subtype indicated to have the potential to infect non-humans (i.e. reported in a three-days suckler calf and a six-year old Aberdeen Angus-cross cow [59]), suggesting possible zoonotic transmission. Based on its dominance in humans and its association with waterborne outbreaks and transmission from animal to humans, it is proposed that IbA10G2R2 merits attention as the most significant *C. hominis* gp60 subgenotype linked to human cryptosporidiosis globally with zoonotic potential [25].

It is interesting to note that genotypes Ia and Id were less commonly found genotypes in humans. Within genotype family Ia, subgenotype IaA12G1R1 has been isolated from human in Japan [60], Nepal [50], Pakistan [46] and Peru [21] whilst another subgenotype IaA21G1R1, has been reported from Egypt, India, Malawi, South Africa and Spain [30], [32], [61]. The results of the present study showed that *C. hominis* infection among HIV patients was caused by *C. hominis* subgenotypes IaA14R1 and IaA18R1. To the best of our knowledge subgenotype IaA18R1 have not been reported previously. Lim et al. [12] reported high diversity of *C. hominis* subgenotypes in HIV individuals, not only IaA14R1 and IbA10G2R2 were detected, but also IdA15R2, IeA11G2T3R1 and IfA11G1R2.

The global gp60 analysis revealed the high genetic richness and variation of gp60 subgenotypes in human and animals [25] but the clinical and epidemiological significance of various *Cryptosporidium* species and subgenotypes in humans are not fully understood yet. However, Cama *et al.* [62] reported that in AIDS patients in Lima, Peru, infections with *C. canis*, *C. felis*, and subgenotype family Id of *C. hominis* were significantly associated with diarrhea, whereas infections with *C. parvum* were significantly associated with chronic diarrhea and vomiting. It was also noted that infections with *C. hominis* Ib subgenotype family were marginally associated with diarrhea and vomiting. In contrast, infections with *C. meleagridis* and Ia and Ie subgenotype families of *C. hominis* were usually asymptomatic [62]. Unfortunately, none of the studies showed the clinical correlation of genotypes and subgenotypes.

The clinical manifestation for Malaysian HIV patients appears to be in contrast with those of Peruvian patients, where infections with *C. parvum* and *C. hominis* subgenotypes were not associated with chronic diarrhea and vomiting. These results demonstrated that different *Cryptosporidium* genotypes and subgenotype families may be linked to different clinical manifestations. Indeed, further detailed investigations are warranted in order to improve understanding of these associations. The occurrence of distinctive multilocus subgenotypes in both *C. hominis* and *C. parvum* populations in Malaysia that can be differentiated from other geographic regions, including the nearby Southeast Asian countries, is notable and requires further investigations. The fact that *C. hominis* from different areas may have an identical gp60 subgenotype but a different microsatellite subtype is also noteworthy, as some *C. hominis* subgenotypes, such as IbA10G2R2, are commonly associated with water-borne outbreaks in industrialized countries. The distinction is important in mapping the transmission of the parasite, especially where tracking infection pathways is necessary, such as in the investigations of environmental and zoonotic transmission.

In conclusion, this study documented the distribution of cryptosporidial species and subgenotypes in HIV infected individuals in Malaysia. Gp60 genotype analysis showed HIV-patients were infected with *C. parvum* IIa and IId genotypes and *C. hominis* Ia and Ib genotypes denoting the possibility of zoonotic as well as anthroponotic transmissions of cryptosporidiosis in these individuals. A comprehensive study analysing clinical manifestations of the gp60 subgenotypes in relation to the distinct microsatellites from different areas need to be studied further. This would promote greater understanding of *Cryptosporidium* epidemiology and various pathogenicities.
